# Long-term Western diet fed apolipoprotein E-deficient rats exhibit only modest early atherosclerotic characteristics

**DOI:** 10.1038/s41598-018-23835-z

**Published:** 2018-04-03

**Authors:** Ida Rune, Bidda Rolin, Jens Lykkesfeldt, Dennis Sandris Nielsen, Łukasz Krych, Jenny E. Kanter, Karin E. Bornfeldt, Pernille Kihl, Karsten Buschard, Knud Josefsen, Johannes Josef Fels, Alan Mortensen, Berit Christoffersen, Rikke Kaae Kirk, Axel Kornerup Hansen

**Affiliations:** 1 Section of Experimental Animal Models, Department of Veterinary and Animal Sciences, Faculty of Health and Medical Sciences, University of Copenhagen, Frederiksberg, Denmark; 2 Metabolic Disease Research, Novo Nordisk A/S, Måløv, Denmark; 3 Department of Food Science, Faculty of Science, University of Copenhagen, Frederiksberg, Denmark; 4 Department of Medicine, UW Medicine Diabetes Institute, University of Washington, Seattle, Washington, USA; 5 Bartholin Institute, Rigshospitalet, København, Denmark; 6 Research Bioanalysis, Novo Nordisk A/S, Måløv, Denmark

## Abstract

In the apolipoprotein E–deficient mouse, the gut microbiota has an impact on the development of atherosclerosis, but whether such correlations are also present in rats requires investigation. Therefore, we studied female SD-*Apoe*^*tm1sage*^ (*Apoe*^−/−^) rats fed either a Western diet or a low-fat control diet with or without gluten, which is known to promote gut microbiota changes, until 20 weeks of age. We hypothesized that the manifestation of atherosclerosis would be more severe in *Apoe*^−/−^ rats fed the Western high-fat diet, as compared with rats fed the low-fat diet, and that atherosclerosis would be accelerated by gluten. Both Western diet-feeding and gluten resulted in significant changes in gut microbiota, but the microbiota impact of gluten was transient. Compared with *Apoe*^−/−^ rats fed a low-fat diet, Western diet-fed *Apoe*^−/−^ rats were heavier and became glucose intolerant with increased levels of oxidative stress. They developed early fatty streak lesions in their aortic sinus, while there was no evidence of atherosclerosis in the thoracic aorta. No conclusions could be made on the impact of gluten on atherosclerosis. Although Western diet-fed *Apoe*^−/−^ rats exhibited a more human-like LDL dominated blood lipid profile, signs of obesity, type 2 diabetes and cardiovascular disease were modest.

## Introduction

In the past 25 years, genetically altered mouse models, such as the apolipoprotein E (ApoE) deficient (*Apoe*^−/−^) mouse, have been studied to understand the underlying pathophysiological mechanisms of cardiovascular disease and the development of therapies^1,2^. The *Apoe* gene is expressed in numerous tissues including brain, liver and adipose tissue^1,3^ and ApoE mediates the binding of chylomicrons and LDL to the LDL-receptor, preventing the accumulation of cholesterol rich particles in the plasma^1,4–8^. Mouse studies have demonstrated that loss of ApoE results in accumulation of triglyceride-rich lipoprotein (TRL) remnants, elevated cholesterol in the blood and the development of atherosclerotic lesions; a condition, which can be accelerated by diets rich in fat and cholesterol^9,10^. Aortic lesions progress to advanced human-like stages, characterized by the presence of necrotic cores, fibrous caps, extracellular matrix components, and cholesterol clefts.

Within recent years the gut microbiota has been accepted as an important player in development of a series of conditions – including cardiovascular disease and insulin resistance^11–13^. Genetic factors and equally important environmental factors such as diet, antibiotic or prebiotic treatments play central roles in establishing and also maintaining an individual’s gut microbiota composition^14–18^. Mice fed diets containing gluten or its active component gliadin differ significantly in their gut microbiota composition from mice fed a gluten free diet^19^. A gluten free diet has a dramatic impact on reducing the incidence of type 1 diabetes^20^ in NOD mice, a less dramatic impact on the development of type 2 diabetes^21,22^, and as we have recently demonstrated, it does not impact development of atherosclerosis in *Apoe*^−/−^ mice^23^. In these mice, the impact of the gut microbiota is implicated by results demonstrating that ampicillin treatment reduces LDL and VLDL cholesterol levels and protects from aortic lesion development^23^. Also the gluten effect on gut microbiota is strong at the age of five weeks, but it seems to disappear at the age of 16 weeks^23^. The size of rats is advantageous, as this facilitates plaque lesion analysis, tissue and blood collection and more extensive plasma analyses. Rodents generally have rapid LDL-clearance, and HDL-cholesterol is the largest of the lipoprotein fractions^1,24,25^, which might explain why rodents do not spontaneously develop atherosclerosis.

Due to limited possibilities of cultivating embryonic stem cells from other species, gene knockout has mostly been performed on mice until a few years ago. Today, the introduction of the nuclease techniques, such as Zinc Finger (ZF)^26^, Transcription Activator-Like Effector Nuclease (TALEN)^27^ and recently Clustered Regularly Interspaced Short Palindromic Repeats (CRISPR/Cas9)^28^ have enabled easy gene deletion in species other than mice. In 2011, scientists at Sigma’s Advanced Genetic Engineering (SAGE) Labs created ApoE gene knockout (*Apoe*^−/−^) rats by the use of ZF technology^29^. Only limited data on *Apoe*^−/−^ rats exist^29,30^. In two previous studies describing *Apoe*^−/−^ rat models^29,30^, very early fatty streak lesions were demonstrated. However, in one of the studies, in which the rats were fed a high fat diet for only 12 weeks, no vascular lesions were detected unless the carotid artery was partially ligated^30^. So, in general there is an obvious need to further validate *Apoe*^−/−^ rats as a model of dyslipidemia, glucose intolerance, changes in gut microbiota, and atherosclerosis and more specifically, to investigate the translational aspects of our previous observations on the relationship between gut microbiota and atherosclerosis in *Apoe*^−/−^ mice in a larger model such as a rat.

**Figure 1:**
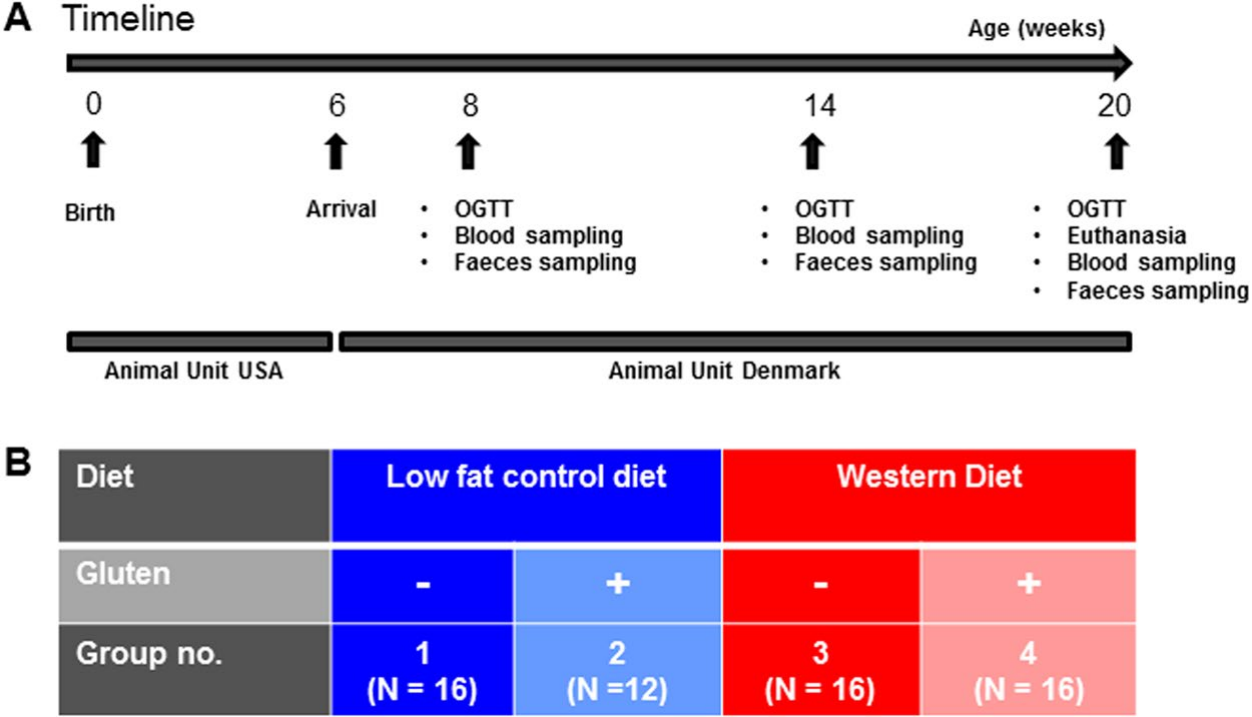
Timeline and Study design. **(A**) Timeline of the study showing time points for Oral glucose tolerance test (OGTT), blood and faeces sampling. (**B)** Animals were divided into four dietary groups; Group LF received a low fat control diet (n = 16 at all time points), group LF + G received a low fat control diet containing 3.5% gluten (n = 16 at week 8, n = 15 at week 14 and n = 14 at week 20; 2 rats were euthanized at week 11 and 19, respectively), group WD received Western Diet (n = 14 at week 8, n = 13 at week 14 and n = 13 at week 20; 3 rats were euthanized at week 6, 7 and 8, respectively), and group WD + G received Western Diet containing 3.5% gluten (n = 8 at all time points (4 rats were euthanized on or before week 7).

The aim of the present study, therefore, was to evaluate the development of atherosclerosis, weight gain, insulin resistance and early signs of liver damage, in *Apoe*^−/−^ rats under two different gut microbiota compositions, i.e. with or without gluten, when fed a Western high-fat diet (WD) compared to a control low-fat diet (LF) for a longer period of time than previous studies (Fig. 1). We hypothesized that gluten would change the gut microbiota composition, that the manifestation of atherosclerosis would be more severe in Western high-fat diet fed compared to low-fat diet fed rats, and that the manifestation of atherosclerosis would be accelerated in gluten-fed rats as compared with rats fed a gluten-free diet.

## Results

### Dietary fat and gluten change gut microbiota composition in *Apoe*^−/−^ rats

**Figure 2:**
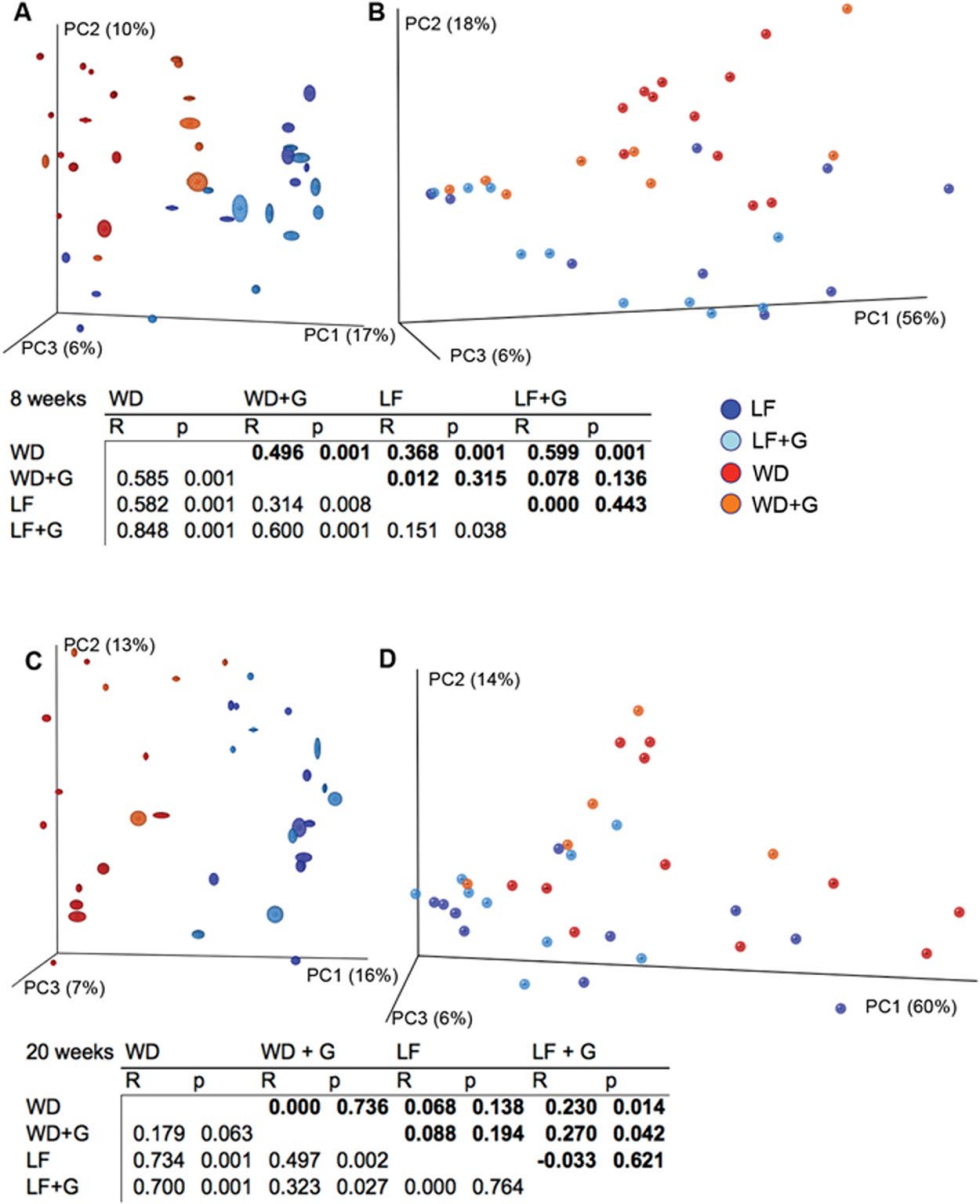
Gut microbiota composition changes in response to Western (WD) or low fat (LF) diet and gluten (**G**) content. (**A**–**D**) PCoA plots generated based on unweighted (**A**) and weighted (**B**) distance metrics visualizing the level of qualitative and quantitative similarities between the gut microbial composition of young (8 weeks of age), and adult rats (20 weeks of age) - unweighted (**C**) and weighted (**D**). Tables below the plots present results of ANOSIM analysis performed for randomly selected unweighted (regular font) or weighted (bold font) rarefied uniFrac distance metrics.

**Figure 3:**
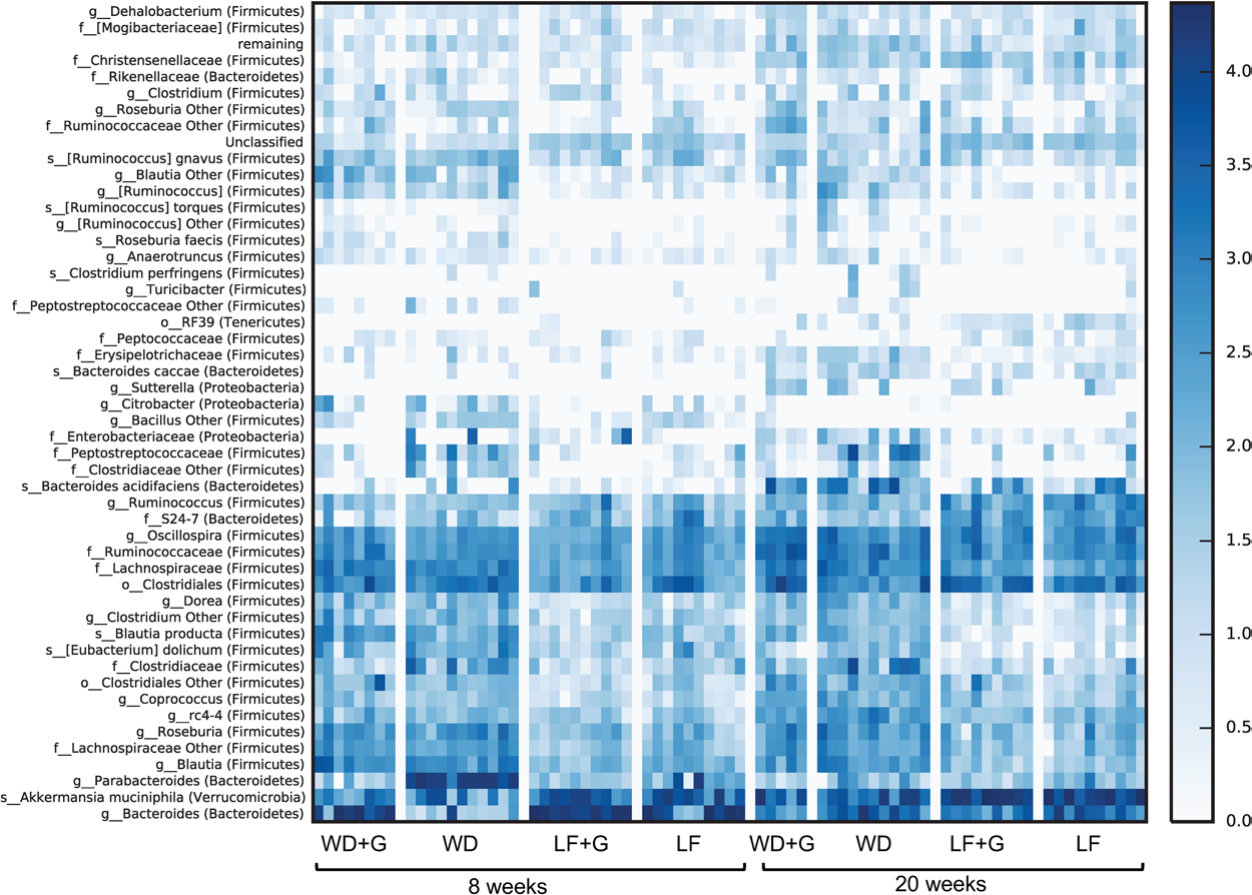
Western Diet (WD) and/or gluten feeding of *Apoe*^−/−^ rats induces changes in composition of low abundant microbiota taxa. Dietary gluten reduced the abundance of the genus *Parabacteroides* (50%, 0.2%, 9%, and 0.2%) in the Western Diet without gluten (WD), Western Diet with gluten (WD + G), low fat control without gluten (LF), and low fat control with gluten (LF + G) category, respectively), the relative abundance of which was increased in eight week old WD-fed compared to LF-fed rats (Bonferroni corrected P = 0.000). In the 20 weeks old animals, the genus *Eubacterium* (greengenes13.8 unofficial name representing the *Erysipelotrichaceae* family) was significantly increased in abundance (Bonferroni corrected P = 0.014) in the WD group (0.4%, 0.1%, 0.03%, and 0.02% in the WD, WD + G, LF, and LF + G category, respectively). No significant qualitative differences in genera distribution between tested categories were found with G-test. Worth noting is it that the number of estimated species in the LF + G group was significantly reduced at eight weeks of age compared to the WD and WD + G categories (P = 0.042 and P = 0.012 respectively; data not shown).

Female *Apoe*^−/−^ rats were divided into four groups, and each group was fed one of four different diets; a gluten-free low-fat diet (LF), a gluten-containing LF diet (LF + G), a gluten-free Western diet (WD) or a gluten-containing WD (WD + G), as shown in Fig. 1A,B. To avoid maternal milk gluten transfer, the experimental diets were fed *ad libitum* to the mothers from approximately 10 days pre-partum, and subsequently to the offspring until 20 weeks of age. Qualitatively, compositional gut microbiota changes in eight week-old rats were demonstrated as a response to both dietary fat (WD vs. LF; P = 0.001 and WD + G vs. LF + G; P = 0.001, un-weighed data, Fig. 2A) and dietary gluten content (WD vs. WD + G; P = 0.001 and LF vs. LF + G; P = 0.038, un-weighed data, Fig. 2A). At 20 weeks of age, clustering could only be demonstrated with respect to the dietary fat content (WD vs. LF; P = 0.001 and WD + G vs. LF + G; P = 0.027, un-weighed data, Fig. 2C). Quantitatively, significant differences as a response to dietary fat content were observed in the eight week-old animals (WD vs. LF; P = 0.001, weighed data, Fig. 2B), and after 20 weeks (WD + G vs. LF + G; P = 0.042, weighed data, Fig. 2D). WD differed significantly from LF + G at both eight and 20 weeks and both quantitatively (P = 0.001 and P = 0.014, respectively) and qualitatively (P = 0.001 and P = 0.001, respectively) (Fig. 2), while WD + G also differed significantly from LF at both eight and 20 weeks, but only qualitatively (P = 0.008 and P = 0.002) (Fig. 2). As revealed by the un-weighted UniFrac distance metrices analysis, there were significant differences among less abundant species, such as *Parabacteroides* and *Eubacterium* (Fig. 3).

### Western diet-fed *Apoe*^−/−^ rats develop small early fatty streaks in the aortic sinus but not in the thoracic aorta

**Figure 4:**
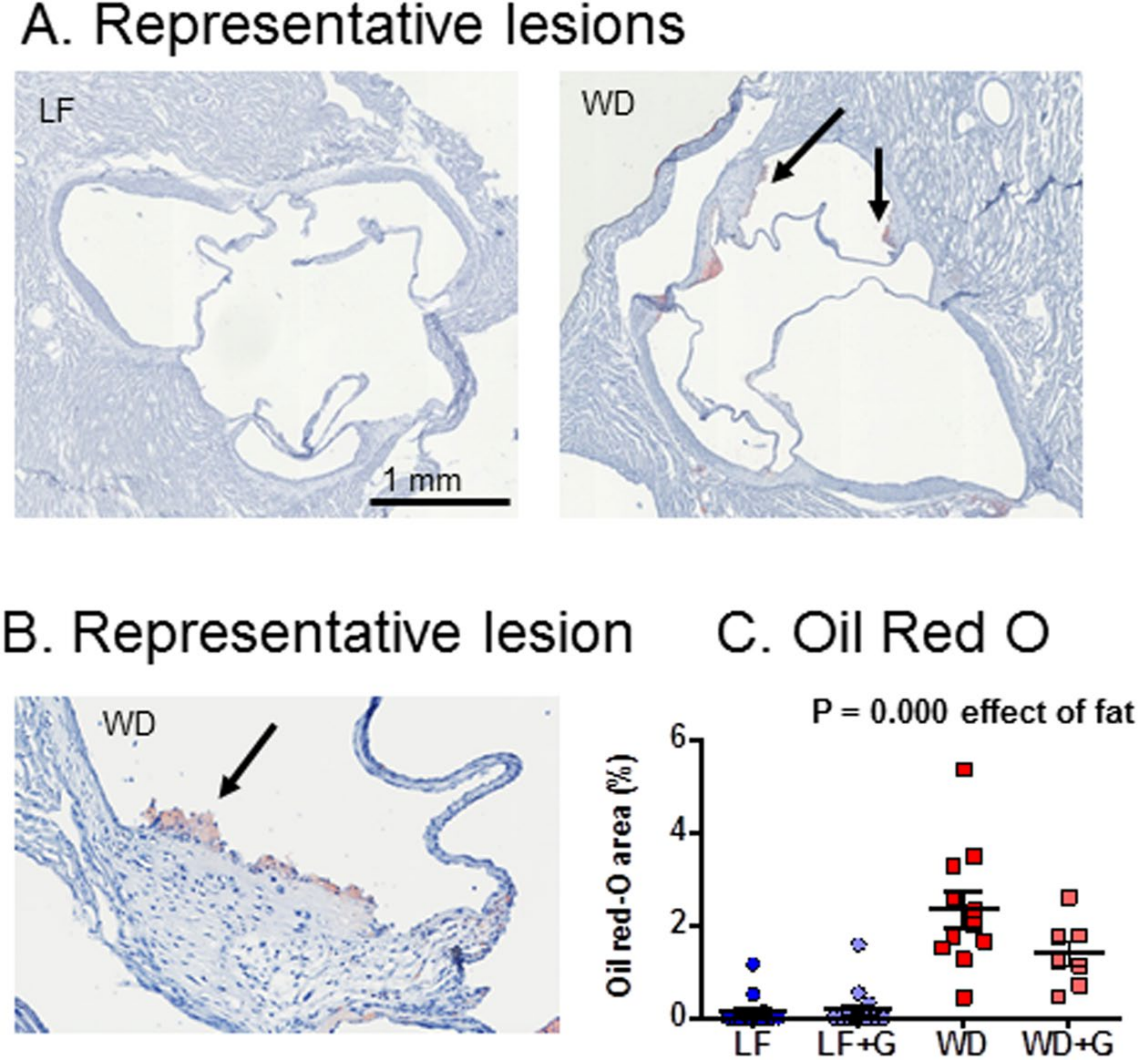
Western Diet (WD)-fed *Apoe*^−/−^ rats exhibit small fatty streak lesions of atherosclerosis limited to the aortic sinus. (**A)** Histological slides of the aortic sinus in an *Apoe*^−/−^ rat fed the LF diet without detectable plaque (40X, Stained with Oil Red O), and *Apoe*^−/−^ rat fed the WD with an early fatty streak-type lesion (40X, Stained with Oil Red O). (**B**) Higher magnification of the fatty streak lesion in A. The lesion is indicated by an arrow. (**C**) Graphical representation of the lesion area measurements at 20 weeks of age showed significantly higher plaque burden in animals fed Western diets (groups 3 and 4) compared with animals fed the low fat control diet. Data are expressed as mean ± SEM. n = 14 in the low fat (LF) and low fat + gluten (LF + G) groups, n = 11 in the Western Diet (WD) group and n = 7 in the Western Diet + gluten (WD + G) group.

By the method described by Tangirala and reviewed by Meir^1,31^, we found evidence of very early fatty streak lesions in the aortic sinus. WD-fed rats exhibited small early fatty streak lesions, whereas no lesions were present in LF-fed rats (Fig. 4A,B). The oil red O-positive area out of total cross-sectional aortic sinus intimal area was significantly lower in LF-fed compared to WD-fed rats, but the effect of WD-feeding was very low compared to previous findings in the *Apoe*^−/−^ mouse at a similar age (P = 0.000; 0.14 ± 0.09% in LF-fed rats, 0.18 ± 0.12% in LF + G-fed rats, 2.34 ± 0.40% in WD-fed rats, and 1.38 ± 0.27% in WD + G-fed rats) (Fig. 4C). Furthermore, the lesions were far less severe^32^. We could not show any accelerating effect of gluten on fatty streak size in WD-fed rats, and although it might seem as if there was an alleviating effect of gluten, power and study design does not allow such conclusions. In the thoracic aortas of 20 week-old rats, no lesions could be detected macroscopically in either WD-fed or LD-fed rats (data not shown), consistent with the previous study on *Apoe*^−/−^ rats by Wei *et al*.^30^. Also, no evidence of increased presence of gene markers of macrophages and monocytes (*Cd68*, *Adgre1*), markers of lipid accumulation (*Abca1*, *Abcg1*), cytokines (*Il1b*, *Il6*, *Tnfa*), chemokines (*Ccl2*), or adhesion molecules (*Icam1*, *Vcam1*) were detected in aortas from WD-fed *Apoe*^−/−^ rats, further supporting the conclusion that even early fatty streak lesions are absent in the thoracic part of aorta at the 20-week time-point (Fig. S1).

### Western diet-fed *Apoe*^*−/−*^ rats have a human-like lipoprotein profile with elevated levels of blood lipids and liver enzymes

**Figure 5:**
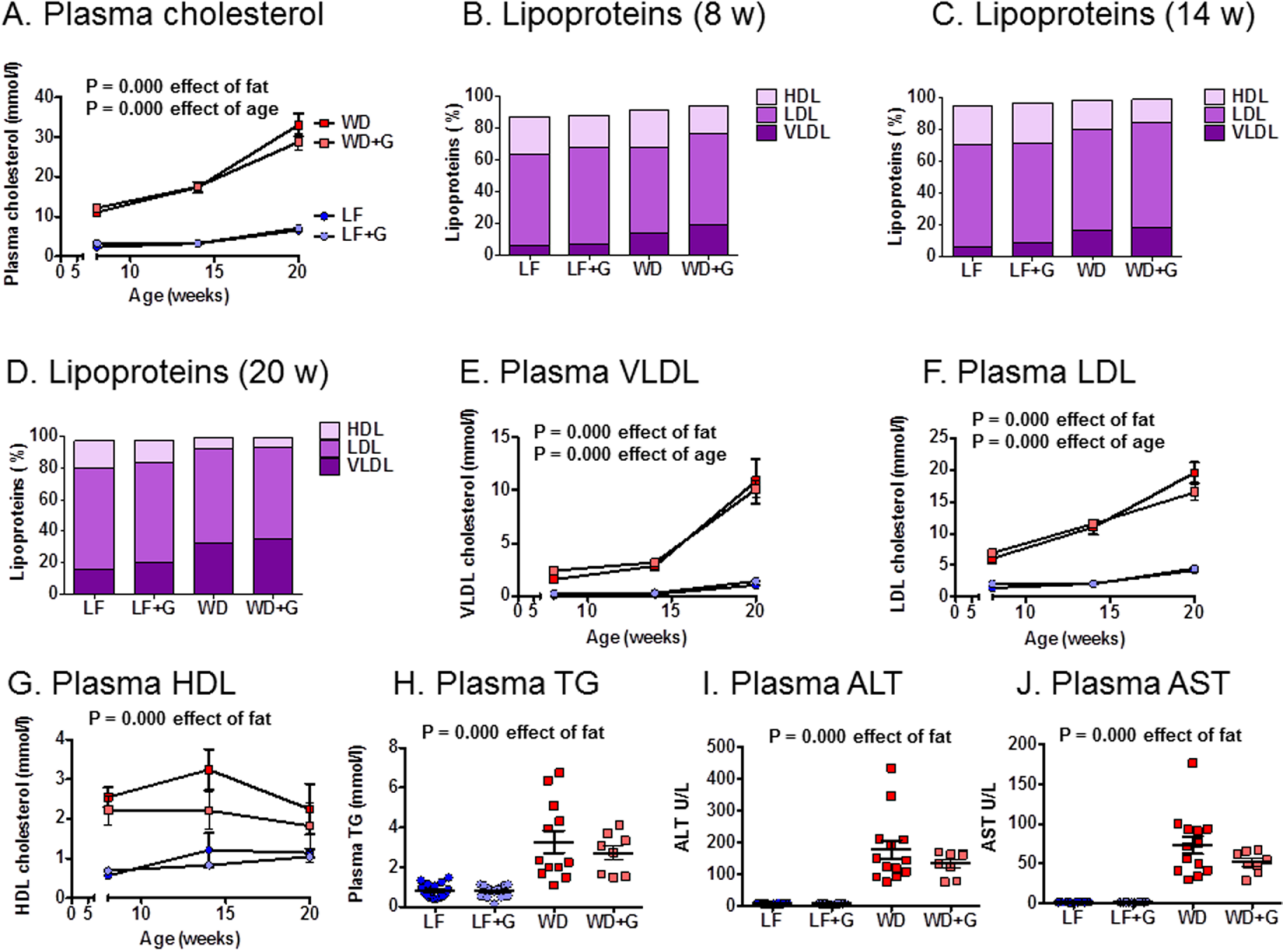
Western diet (WD)-fed *Apoe*^−/−^ rats exhibit elevated blood lipid levels and human-like lipoprotein profiles. (**A**) At 8, 14 and 20 weeks of age low fat diet (LF) fed groups had significantly lower total plasma cholesterol levels than Western Diet (WD) fed groups (n = 4/group). (**B–D)** Graphs showing the percentagewise distribution of the lipoprotein fractions HDL, LDL and VLDL (n = 4/group). See also Table 1. (**E–G)** Data points for VLDL, LDL and HDL cholesterol represent four samples, each consisting of pooled plasma from two rats. There was a significant impact of diet on VLDL and LDL at 8 weeks (P = 0.000 and P = 0.026, respectively), on VLDL also at 14 weeks (P = 0.000) and 20 weeks (P = 0.000) and on HDL at 14 weeks (P = 0.000) and 20 weeks (P = 0.000). (**H**) Triglyceride levels were measured at 20 weeks of age and showed a significant difference between the groups related to dietary fat content (n = 16 in the LF and LF + G groups, n = 12 in the WD group, n = 8 in the WD + G group). (**I,J**) The liver enzymes ALT and AST were both significantly elevated in the WD compared to LF groups (n = 16 in the LF group, n = 14 in the LF + G group, n = 13 in the WD group, n = 8 in the WD + G group). Gluten did not have any significant influence on blood lipids and liver enzymes. Data are expressed as mean ± SEM.

Total plasma cholesterol levels were significantly higher in the WD-fed rats at 8, 14 and 20 weeks of age (Fig. 5A) compared to LF-fed rats (P = 0.000). A plasma cholesterol level of 30–35 mmol/L after 20 weeks on WD is similar to levels in TALEN-generated *Apoe*^−/−^ rats fed a high fat diet for 12 weeks^30^. In the present study, the *Apoe*^−/−^ rats had human-like plasma profiles with LDL-fractions not dropping below 50% of the total plasma cholesterol at any time point regardless of diet (Fig. 5B–D and Table 1). Both total VLDL and the relative level of VLDL were significantly increased after WD feeding as compared to LF feeding (P = 0.000) (Fig. 5B–E). As expected, total LDL levels (Fig. 5F) were significantly increased with WD feeding (P = 0.000), but the relative levels of LDL were stable between experimental groups. Furthermore, even though total HDL levels were significantly increased in WD-fed rats (P = 0.000) (Fig. 5G), the relative contribution of HDL was reduced with WD feeding, especially later on during the study (Fig. 5B–D, Table 1). Consistent with elevated VLDL levels, WD-feeding also resulted in significantly elevated plasma triglyceride levels (P = 0.000) (Fig. 5H). The addition of gluten to the diets had no effect on lipid levels. WD-fed compared to LF-fed rats had significantly higher plasma levels of the liver enzymes ALT and AST (P = 0.000) (Fig. 5I,J).

**Table 1:** Lipoprotein cholesterol fractions either expressed as percentage of total plasma cholesterol or as absolute values (mmol/l).

	LF	LF + G	WD	WD + G	P value
	8 Weeks				
HDL%	23.6 ± 1.3	20.7 ± 1.4	23.1 ± 2.7	18.0 ± 2.8	0.545
HDL mmol/l	0.6 ± 0.1	0.7 ± 0.1	2.5 ± 0.2	2.2 ± 0.4	
LDL%	58.1 ± 1.7	60.7 ± 2.1	53.8 ± 1.6	56.8 ± 1.0	0.026
LDL mmol/l	1.4 ± 0.2	2.1 ± 0.5	6.0 ± 0.7	6.9 ± 0.4	
VLDL%	5.8 ± 0.5	6.9 ± 1.0	14.4 ± 1.1	19.6 ± 3.2	0.000
VLDL mmol/l	0.1 ± 0.0	0.2 ± 0.1	1.6 ± 0.1	2.4 ± 0.4	
	**14 Weeks**				
HDL%	24.7 ± 0.8	25.4 ± 1.2	18.4 ± 2.0	14.9 ± 0.5	0.000
HDL mmol/l	1.2 ± 0.4	0.8 ± 0.0	3.2 ± 0.5	2.2 ± 0.5	
LDL%	64.3 ± 1.5	62.6 ± 1.9	63.1 ± 1.9	65.9 ± 1.5	0.565
LDL mmol/l	2.1 ± 0.2	2.1 ± 0.1	11.0 ± 1.1	11.6 ± 0.4	
VLDL%	5.8 ± 0.6	8.9 ± 1.2	16.8 ± 2.7	18.2 ± 0.6	0.000
VLDL mmol/l	0.2 ± 0.0	0.3 ± 0.0	2.9 ± 0.4	3.2 ± 0.2	
	**20 Weeks**				
HDL%	17.4 ± 0.4	14.4 ± 1.3	7.1 ± 2.1	6.1 ± 1.7	0.000
HDL mmol/l	1.1 ± 0.1	1.0 ± 0.1	2.2 ± 0.6	1.8 ± 0.6	
LDL%	63.4 ± 1.6	62.5 ± 3.3	59.3 ± 2.8	57.5 ± 0.4	0.071
LDL mmol/l	4.2 ± 0.5	4.5 ± 0.4	19.6 ± 1.6	16.5 ± 1.2	
VLDL%	16.1 ± 2.7	20.5 ± 4.7	32.5 ± 4.2	35.2 ± 1.4	0.000
VLDL	1.0 ± 0.2	1.4 ± 0.3	10.9 ± 2.1	10.1 ± 0.8	

LF: Low fat control diet, LF + G: Low fat control diet with added gluten, WD: Western Diet, WD + G: Western Diet with added gluten. Data are presented as mean percentages ± SEM. The listed *p*-value is calculated by two-way ANOVA with source of variation and is the *p*-value describing the difference due to fat and cholesterol content in the diet. There was no difference due to gluten. Significant *p*-values are marked in bold; n = 4/group.

### Western diet-fed *Apoe*^−/−^ rats gain more weight than low fat-fed *Apoe*^−/−^ rats and are glucose intolerant

**Figure 6:**
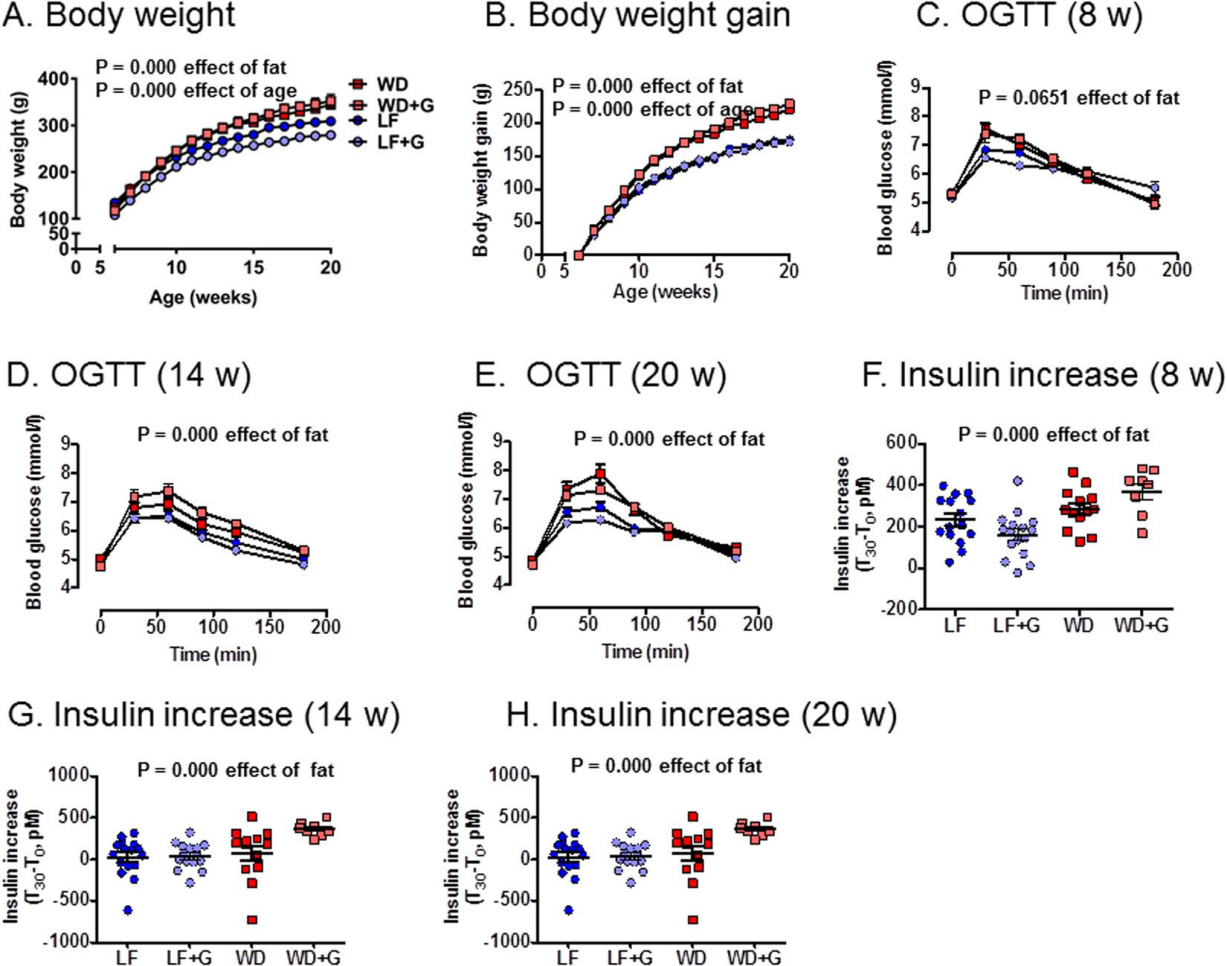
Western Diet (WD)-fed *Apoe*^−/−^ rats gain more weight and are glucose intolerant. (**A**) Dietary fat content related weight differences (Area under curve (AUC)). Low fat (LF) (n = 16), low fat + gluten (LF + G) (n = 14), Western Diet (WD) (n = 13) and Western Diet + Gluten (WD + G) (n = 8). (**B)** Dietary fat content weight gain differences (AUC). (**C**) Oral glucose tolerance tests (OGTT) at 8 weeks did not differ significantly over all (P = 0.0651). (LF (n = 16), LF + G (n = 15), WD (n = 14), WD + G (n = 8)). (**D,E)** OGTTs at 14 and 20 weeks differed significantly related to dietary fat content *(*P = 0.000 and P = 0.000 respectively). Week 14: LF (n = 16), LF + G (n = 15), WD (n = 13), WD + G (n = 8); Week 20: LF (n = 16), LF + G (n = 14), WD (n = 13), WD + G (n = 8). Gluten did not influence OGTTs. (**F–H)** Δ_Insulin_ (*t* = 30 minutes − *t* = 0 minutes) differed significantly between groups related to dietary fat content at all three time points (LF (n = 15), LF + G (n = 16), WD (n = 13), WD + G (n = 8)). Data are expressed as mean ± SEM.

WD-fed rats exhibited a modest, albeit significant, body weight gain, as compared to LF-fed rats (P = 0.000), while dietary gluten did not affect weight gain (Fig. 6A,B). Consistent with these observations, WD-fed rats showed an overall impaired glucose tolerance at 14 and 20 weeks of age (P = 0.000), as compared with the LF-fed rats (Fig. 6C–E), and at 8, 14 and 20 weeks of age, their rise in insulin levels during the first 30 minutes of OGTTs was higher (P = 0.000) (Fig. 6F–H). However, this did not result in elevated levels of glycated hemoglobin (HbA1c) (Fig. S2).

### Western diet-fed *Apoe*^−/−^ rats exhibit elevated markers of oxidative stress

**Figure 7:**
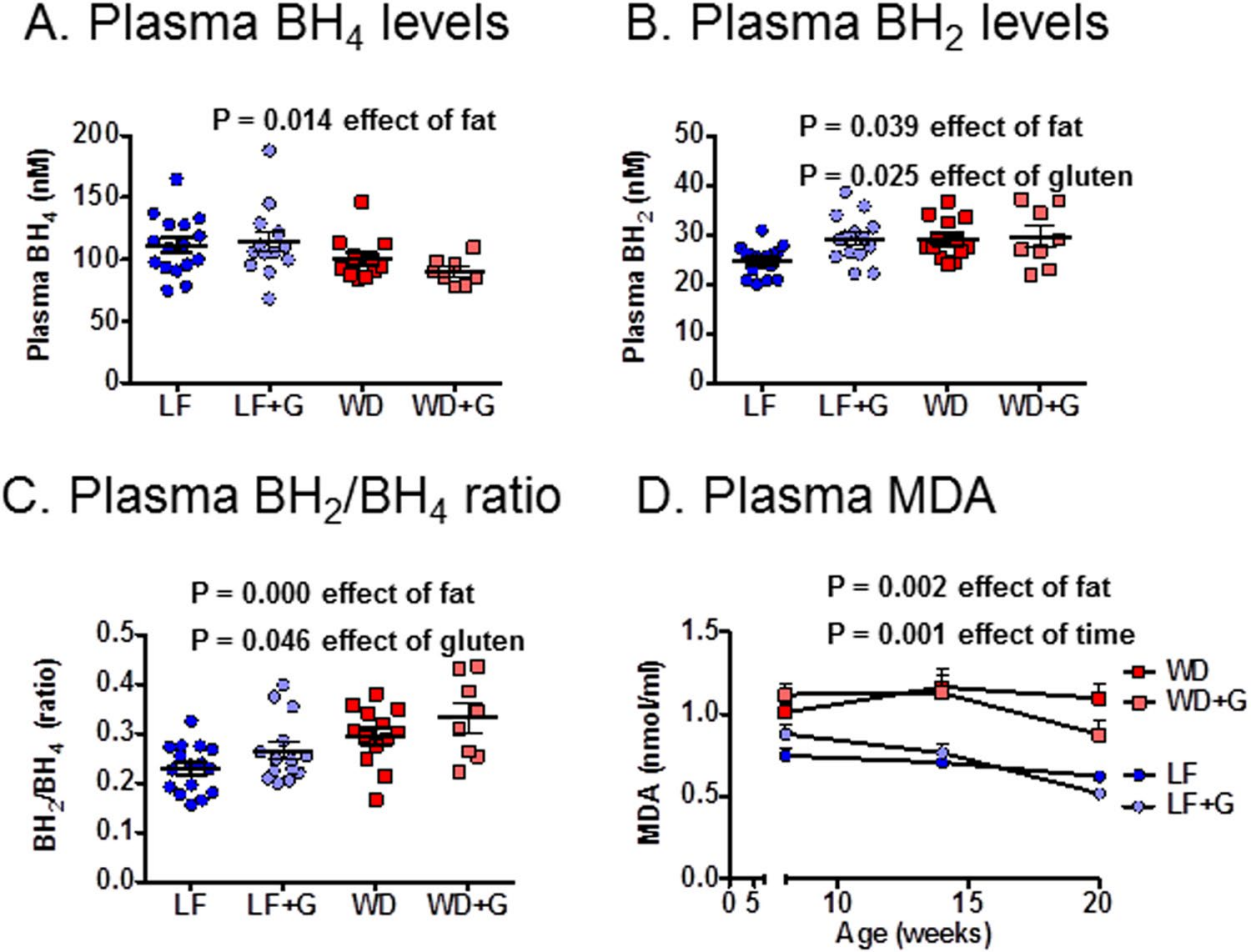
Biomarkers of oxidative stress are increased in Western (WD) compared to low fat (LF) diet-fed *Apoe*^−/−^ rats. (**A,B**) At 20 weeks of age tetrahydrobiopterin (BH_4_) levels in plasma showed significant difference between the groups due to fat content in the diets, whereas dihydrobiopterin (BH_2_) showed no group differences (n = 16 in the LF group, n = 14 in the LF + G group, n = 13 in the WD group and n = 8 in the WD + G group). (**C)** The BH_2_/BH_4_ ratio also showed a significant difference between the groups due to fat content in the diets. (**D)** At all three time points a difference in plasma levels of malondialdehyde (MDA) between the groups due to fat content in the diets could be demonstrated (data from all time points from n = 16 in the LF group, n = 14 in the LF + G group, n = 13 in the WD group and n = 8 in the WD + G group).

Oxidative stress might be elevated by WD feeding, so two markers of oxidative stress were measured. In the WD-fed rats, lower levels of reduced BH_4_ compared to its oxidation product BH_2_ may be expected as a result of increased oxidative stress. Supporting this hypothesis, we found that the LF-fed animals had significantly higher plasma BH_4_ and lower BH_2_ levels compared to the WD-fed animals (BH_4_: P = 0.014; BH_2_ P = 0.039; BH_2_/BH_4_ P = 0.000), and also that gluten increased the BH_2_ levels and thereby the BH_2_/BH_4_ ratio significantly (BH_2_ P = 0.025; BH_2_/BH_4_ P = 0.046) (Fig. 7A–C). Malondialdehyde (MDA) is an end-product of lipid oxidation and has been detected in oxidized LDL^33^. Plasma MDA levels were significantly higher in WD-fed compared to LF-fed rats, indicating elevated oxidative stress (P = 0.002) (Fig. 7D). Gut microbiota composition correlated with MDA levels in both young and adult rats in this study. Thus, in the eight weeks old rats, genera *Roseburia*, *Coprococcus*, and two unclassified genera belonging to the *Lachnospiraceae* family, correlated positively with the MDA level [P = 0.007 (*r* = 0.572), P = 0.009 (*r* = 0.562), P = 0.010 (*r* = 0.559) and P = 0.040 (*r* = 0.512), respectively, Bonferroni corrected]. In the 20 weeks old rats, genus *Dorea*, *Clostridium* and an unknown genus belonging to the *Erysipelotrichaceae* family also correlated positively with the MDA level [P = 0.002 (*r* = 0.638), P = 0.010 (*r* = 0.587) and P = 0.009 (*r* = 0.593) respectively, Bonferroni corrected]. The increased oxidative stress did not correlate with plasma levels of IL-1α, CCL2, IL-4, IFNγ, and TNF-α, but these cytokine levels were below detection in most animals (Fig. S3).

## Discussion

In the present study, we show that female *Apoe*^−/−^ rats exhibit early lesions of atherosclerosis in the aortic sinus, when fed a Western high-fat diet, as compared with a low fat diet. In relation to dietary gluten, the study design only allowed us to conclude that there was a minor impact on the oxidative stress marker BH_2_, and an early life change in gut microbiota composition, which is in agreement with previous data in *Apoe*^−/−^ mice^23^. The abundance of *Parabacteroides* was decreased by gluten feeding at 8 weeks. This species is one of the most abundant genera of bacteria in the human gastrointestinal tract^34^, it is increased by prenatal exposure to metformin^35^, and it has been shown to be reduced in abundance in patients with irritable bowel syndrome or ulcerative colitis^36^. However, although the reduced abundance of *Parabacteroides* as a response to gluten feeding fits the expectation that gluten feeding might exert pro-inflammatory effects, it is not possible from our data to conclude whether gluten might have had effects on early or more advanced atherosclerosis.

The abundance of *Erysipelotrichaceae* was increased after 20 weeks of Western diet feeding in our study, and this has also been observed as a response to high fat feeding in mice^37^, while prenatal exposure to metformin has been shown to reduce *Erysipelotrichaceae*^35^. Different microbial taxa correlated with MDA at different ages, which emphasizes findings in previous studies showing age differences in the gut microbiota composition and also the presence of a window of opportunity for altering the gut microbiota^18,38,39^.

The most promising characteristics of WD-fed *Apoe*^−/−^ rats seem to be the more human-like LDL dominated lipid profile, the increase in liver enzymes indicating a potential for developing steatohepatitis as a late complication, and obesity, which is linked to an increased oxidative stress level. The presence of markedly elevated LDL, VLDL and elevated triglycerides in female *Apoe*^−/−^ rats is consistent with an earlier study in male rats^30^.

On the other hand, the manifestations of obesity, type 2 diabetes and atherosclerosis are modest in this new rat model, although it is possible that atherosclerosis severity would have been different in male rats. Compared to results from *Apoe*^−/−^ mice, a weight gain of less than 15% in WD-fed rats compared to LF-fed rats is at the low end^40,41^. HbA1c remains unaffected, and at any time point the increased glucose intolerance is counteracted by an increased insulin response, so after 20 weeks of WD-feeding, these rats still could not be characterized as type 2 diabetic, but rather represented a pre-diabetic state. Also, cardiovascular changes were observed only as fatty streak lesions in the aortic sinus with both lower area and less severity, i.e. the mean percentage of the lesions of WD fed rats was about 2.5% of the total cross-sectional aortic sinus intimal area after 20 weeks, while in previous observations in *Apoe*^−/−^ mice it was about 8%^23^. This indicates that a longer study may be required to induce larger and more advanced lesions in *Apoe*^−/−^ rats. Therefore, the present study gives an unfavorable impression of the *Apoe*^−/−^ rat as a model to investigate atherosclerosis, as compared with *Apoe*^−/−^ mice. The *Apoe*^−/−^ rat is on the Sprague-Dawley (SD) background. Inter-colony genetic differences occur within the same stock of rodents^42–45^, and also gut microbiota differences, which may account for phenotypical differences^38^, exist between populations^46^. This makes it impossible to select a proper wild-type control, if the exact background stock, as in this case, is unavailable from the same vendor facility. Schemmel *et al*. investigated dietary obesity in seven different rat strains and found that high-fat-fed SD rats gained only approximately 25% more body weight than grain-fed controls of the same age and sex, but the increase in body weight could partially be ascribed to an increase in body fat^47^. Levin *et al*. found that only around 50% of SD rats respond to high fat diet by becoming obese whereas the remainders are resistant to diet-induced obesity^48^. The group also demonstrated the ability to overcome this issue by selectively breeding the highest and lowest responders leading to two phenotypically different groups of animals by the F_3_ generation with only 2% and 14% overlap in weight in females and males, respectively^48^. So, not only is it generally problematic to use an outbred background for a genetically modified model, but in this case, the specific choice of SD as the background may also be unfavourable.

In conclusion, the *Apoe*^−/−^ rat responds to 20 weeks of WD feeding by developing dyslipidaemia characterized by high levels of LDL and VLDL cholesterol in plasma, glucose intolerance, and oxidative stress. Atherosclerotic lesions are small, i.e. early fatty streaks limited to the aortic sinus, and in general, all disease symptoms are rather modest and, as in *Apoe*^−/−^ mice, they are not alleviated by a gluten-free feeding. The *Apoe* mutation should be backcrossed to one or several inbred strains to elucidate whether a more disease-prone phenotype could be achieved, and to allow comparison with a proper wild-type control.

## Methods

### Animals and study design

Experiments were in accordance with the EU directive 2010/63/EU and the Danish Animal Experimentation Act (LBK 1306 from 23/11/2007 with 2011 amendments) and approved by the Animal Experiments Inspectorate, Ministry of Environment and Food, Denmark. Sixty female SD-*Apoe*^*tm1sage*^ (*Apoe*^−/−^) rats from Sigma Advanced Genetic Engineering (SAGE) Labs (Boyertown, USA) were randomly divided into four groups housed as 3–5 individuals per cage in a 12-hour light interval at room temperature (Novo Nordisk A/S, Denmark) (Fig. 1A). To avoid gliadin transfer with maternal milk, the experimental diets (all Research Diets Inc., New Brunswick, NJ, USA) were fed *ad libitum* to the mothers from approximately 10 days pre partum, and subsequently to the offspring until 20 weeks of age. Group LF was fed a gluten-free low fat control diet (Cat. #98121701, 10% energy from fat and no cholesterol); group LF + G was fed similarly (Cat. #98121704) with 3.5% added gluten (Cat. #G5004, Sigma, St. Louis, USA), i.e the concentration in ordinary barley-based rodent chow^49^; group WD was fed a gluten-free Western Diet (Cat. #D12079B, 41% energy from fat and 0.21% cholesterol); and group WF + G was fed similarly with added gluten (Cat. #D11061501–2) (Fig. 1B). LF diet was iso-calorimetric with the LF + G diet and the WD diet was iso-calorimetric with the WD + G diet. Prior to euthanasia by cardiac perfusion with a cooled physiological saline solution, animals were anaesthetized with a Hypnorm/Dormicum mixture (1:1:2 water solution, Vetapharm Ltd, Sherburn in Elmet, Leeds, UK; Roche A/S, Hvidovre, Denmark) subcutaneously. Animals were subjected to daily visual controls, and a veterinarian was consulted if signs of illness or misthriving appeared, leading to the euthanasia of nine animals. Two rats were euthanized in the LF + G group due to an eye injury or an extensive wound, while three rats in the WD group and four rats in the WD + G group died due to acute respiratory distress, i.e. there was a higher incidence of fatal acute respiratory distress in the WD-fed groups (P = 0.003), while this was not significantly influenced by gluten. However, it is questionable whether the mortality was due to the WD feeding, as all of these rats died very early in the study (see legend of Fig. 1).

### Study parameters

Animals were weighed weekly. Oral glucose tolerance tests (OGTT) were performed at 8, 14 and 20 weeks of age using a glucose solution (2 g/kg, Fresenius Kabi, Copenhagen, Denmark; concentration 500 g/l) following four hours of fasting (Fig. 1A). Blood glucose levels were measured at *t* = 0, 30, 60, 90, 120 and 180 minutes by transferring 10 µl whole-blood to 500 µl Glucose/Lactate System Solution (EKF Diagnostic GmbH, Barleben, Germany) followed by analysis using a Biosen glucose auto analyser (Eppendorf, Hamburg, Germany) as per the manufacturer’s instructions. Insulin levels were measured during the OGTT’s at *t* = 0 and *t* = 30 minutes on EDTA-stabilized plasma samples using the Ultra-sensitive rat insulin ELISA kit (Crystal Chem, Downer’s Grove, USA) with the modification that in-house rat insulin standards, prepared using heat-treated rat plasma, were used.

Total plasma cholesterol (TPC) and cholesterol in lipoprotein fractions were measured on plasma samples obtained in relation to fasting and OGTT (Department of Pathology/Lipid Sciences, Wake Forrest University School of Medicine (Winston-Salem, NC, USA)). TPC levels were measured using colorimetric enzymatic assays as previously described^50–52^. For lipoprotein measurements, an aliquot of plasma containing approximately 20 µg of TPC was diluted in phosphate buffered saline (PBS) into a final volume of 400 µl. After centrifugation to remove protein precipitates, samples were injected onto a Superose 6 HR 10/30 (Amersham Pharmacia, NJ, USA) chromatography column subsequently run at 0.4 ml/min. The signal was integrated using Chrom Perfect Spirit Software (Justice Laboratory Software, NJ, USA). VLDL-, LDL-, and HDL-cholesterol were determined by multiplying the TPC concentration by the cholesterol percentage within the elution region for each lipoprotein class. For TPC and lipoprotein measurements plasma samples were pooled; two animals per pool and four pools corresponding to a total of eight animals per group were measured. Triglyceride (TG) and the liver enzymes ALT and AST were measured at 20 weeks of age, whereas total plasma cholesterol (TPC) levels and lipoprotein fractions were measured at 8, 14 and 20 weeks of age. TG, ALT and AST were measured on a Hitachi 912 analyser (Roche A/S Diagnostics, Mannheim, Germany) according to the manufacturer’s instructions.

Malondialdehyde (MDA) was assessed in plasma as an index of lipid oxidation by high-performance liquid chromatography (HPLC) as previously described^53^. Furthermore, tetrahydrobiopterin (BH_4_) providing reducing equivalents for eNOS^54^ as well as its oxidation products dihydrobiopterin (BH_2_) and biopterin were measured in plasma as indicators for vascular oxidative stress using HPLC with fluorescence detection employing iodine oxidation as described by Fukushima and Nixon^55^. To prevent *ex vivo* oxidation when measuring biopterins, 1 ml of whole blood was drawn with a K_3_-EDTA flushed syringe and immediately added to a microcentrifuge tube containing 25 µl of 2.5% (w/v) dithioerythriol (DTE) in Milli-Q (18.2 MΩ) water^56^. The sample was then centrifuged (4 °C; 15000 × g; 1 minute) to obtain plasma, which was immediately stored at −80 °C.

After trans-cardiac perfusion with 5 × 10 ml 0.9% NaCl, the heart and 1–2 mm of the aortic sinus were fixed in 10% buffered formalin for 24 hours and transferred to 20% sucrose in phosphate buffered saline (PBS) for 24 hours. The hearts were trimmed by removing the lower part of the heart with the cutting axis perpendicular to the aortic root. The trimmed hearts were placed in a cryo mold with the cut surface at the bottom of the mold, cryo fixated in OCT compound (Tissue-Tek, Sakura Fineteck, Værløse, Denmark) and frozen on dry ice. Sections were cut and discharged until the appearance of the aortic valve. Hereafter, all sections were cut at 10 µm thickness and were collected and placed on numbered glass slides (SuperFrost Plus, Hounisen, Denmark), two on each glass slide with a distance of 150 µm between the sections. Sections were stained with oil red O (Sigma-Aldrich, MO, USA) and scanned on a digital slide scanner (Hamamatsu NanoZoomer 2.0 HT, Japan) at a magnification of 20X. The oil red O stained area was measured using an image analysis tool (Visio-morph) and expressed as the mean of the oil red O-positive area out of total cross-sectional aortic sinus intimal area.

Gut microbiota profiles were determined using tag-encoded 16S rRNA gene MiSeq (Illumina, CA, USA) sequencing. Cellular DNA was extracted using a QIAamp DNA Stool Mini Kit (Qiagen, Hilden, Germany) according to the manufacturer’s instructions, but with the addition of an initial bead-beating step (FastPrep, MP Biomedicals, CA, USA) to increase cell lysis. Extracted DNA was stored at −40° until analysis. Amplicons (~460 bp) including the V3 and V4 regions of the 16S rRNA gene were amplified using primers containing overhangs compatible with Nextera Index Kit (Illumina, CA, USA), tagged and sequenced (250 bp paired end) as previously described^57^.

### Statistical analyses

Data were analysed by two-tailed, two-way ANOVA in either GraphPad Prism version 6 or 7 (GraphPad Software, CA, USA; OGTT (area under the curve (AUC)), weight curves (AUC), insulin levels, blood lipid levels, lipoprotein fractions, histological data, HbA1c, plasma cytokines and gene expression levels in the aortic arch) or Minitab 17 (Minitab Ltd, Coventry, UK; oxidative stress markers (MDA and biopterins). Mortality rates were compared by Fisher’s Exact Test in GraphPad Prism version 7. Data were considered significant when having a *p*-value of less than 0.05 and P = 0.000 was set as lowest reported p-value. Values below detectable limits (insulin and cytokine measurement kits) were given the value of half the lover limit of quantification (½LLOQ).

Gut microbiota data were analysed as previously described^57^. In brief, the raw dataset was trimmed using CLC Genomic Workbench (CLC bio, Aarhus, Denmark) with merging of overlapped reads and using the Quantitative Insight Into Microbial Ecology (QIIME) tool (version. 1.7.0; Open source software)^58^. USEARCH was used to purge the dataset from chimeric reads, while the usearch61 method was used for Operational Taxonomic Units (OTU) selection^59^ with the Greengenes (version 12.10) 16S rRNA gene database as reference^60^. Principal Coordinate Analysis (PCoA) plots were generated with the Jackknifed Beta Diversity workflow based on 10 distance metrics calculated using 10 subsampled Operational Taxonomic Units (OTU) tables taking 85% of the sequence number within the most indigent sample for each jackknifed subset. Analysis of similarities (ANOSIM) was used to evaluate group differences using weighted and unweighted uniFrac distance metrics generated based on rarefied (25,000 reads per sample) OTU tables. Alpha diversity measure expressed with an observed species (sequence similarity 97% OTUs) value was computed for rarefied OTU tables (25,000 reads per sample) using alpha rarefaction workflow. Testing on the differences in alpha diversity was conducted using t-test employing the non-parametric (Monte Carlo) method (999 permutations) implemented in the compare alpha diversity workflow. Determining differences in taxa composition between categories was performed using otu_category_significance.py script implemented in QIIME 1.7.0. The G test of independence (G-test) and ANOVA determining respectively: qualitative (presence/absence) and quantitative (relative abundance) association of OTUs in the given category were calculated based on 999 subsampled OTU-tables rarefied to an equal number of reads (25,000 per sample).

Correlations between the metabolic parameters, biomarkers of oxidative stress, atherosclerosis quantification measures, and taxa relative abundance summarized to the genus level in samples verified at 8 or 20 weeks of age were tested with the Pearson’s product-moment correlation coefficient implemented in the otu_category_significance.py script (QIIME 1.7.0). Testing was performed based on 999 rarefied OTU tables unified to an equal number of reads per sample (25,000).

### Equipment and settings for figures

All statistical graphs (Figs 4–7) were computed by GraphPad Prism versions 6 and 7, while microbiota graphs (Figs 2 and 3) were computed by QUIIME. All Figures, except for Fig. 3, which was based upon a PDF file, were plotted into Power Point (Microsoft, WA, USA), fitted in size and appearance. Subsequently all figures were saved as TIFF images. The images in Fig. 4 have not been edited in other forms of software.

### Data availability

Data and associated protocols are stored on the University of Copenhagen’s backup servers, and can be made available for other non-commercial research groups along with any remaining study materials, as far as still in stock, by communication with the corresponding author.

## Electronic supplementary material

Supplementary Figures
