# Supplementary material for: Long-term Western diet fed apolipoprotein E-deficient rats exhibit only modest early atherosclerotic characteristics
Source: Sci Rep. 2018 Apr 3;8:5416. doi: 10.1038/s41598-018-23835-z (PMC5882891; doi:10.1038/s41598-018-23835-z)
Supplement: Supplementary file 1 — Supplementary Figures [file 41598_2018_23835_MOESM1_ESM.pdf]

Supplementary figures

Long-term Western diet fed apolipoprotein E-deficient rats exhibit only modest early atherosclerotic characteristics

Ida Rune[1], Bidda Rolin[6], Jens Lykkesfeldt[1], Dennis Sandris Nielsen[3], Łukasz Krych[3], Jenny E. Kanter[4], Karin E. Bornfeldt[4], Pernille Kihl[1], Karsten Buschard[5], Knud Josefsen[5], Johannes Josef Fels[6], Alan Mortensen[1], Berit Christoffersen[2], Rikke Kaae Kirk[2] and Axel Kornerup Hansen[1*]

[1]Section of Experimental Animal Models, Department of Veterinary and Animal Sciences, Faculty of Health and Medical Sciences, University of Copenhagen, Frederiksberg, Denmark
[2]Metabolic Disease Research, Novo Nordisk A/S, Måløv, Denmark
[3]Department of Food Science, Faculty of Science, University of Copenhagen, Frederiksberg, Denmark
[4]Department of Medicine, UW Medicine Diabetes Institute, University of Washington, Seattle, Washington, USA
[5]Bartholin Institute, Copenhagen Biocenter, København, Denmark
[6]Research Bioanalysis, Novo Nordisk A/S, Måløv, Denmark

[*]Corresponding author
Email: akh@sund.ku.dk

S1 Figure. Western diet-feeding does not result in increased gene expression of markers of macrophages or inflammation in *Apoe*$^{-/-}$ rats. Thoracic aortas were placed in RNA*later*® (#R0901, Sigma, St. Louis, MO 63103, USA) and kept at -80°C. They were then analysed by Real-Time Quantitative PCR as previously described by Kanter et al [75]. *Rn18s* was used as positive control. The following genes were analysed: *Abca1*, *Abcg1*, *Acsl1*, *Ccl2*, *Cd68*, F4-80, *Icam1*, *Il1b*, *Il6*, *Tnfa* and *Vcam1* (**A-L**). Primer sequences can be obtained on request. All data were analysed by Kruskal-Wallis test with Dunn's multi-comparison test (LF as control group). There were no significant differences between the groups (n=16 in the LF group, n=14 in the LF+G group, n=13 in the WD group and n=8 in the WD+G group).

S2 Figure. Glycated haemoglobin is not altered by Western diet-feeding in *Apoe*$^{-/-}$ rats. HbA1c was measured three times during the study; when the animals were 8, 14 and 20 weeks of age (n=16 in the LF group, n=14 in the LF+G group, n=13 in the WD group and n=8 in the WD+G group). Whole-blood samples were collected from the tail vein and stabilized in Hemolyzing Reagent (Roche/Hitachi, Mannheim, Germany) before being analysed on a Cobas 6000 analyser according to the manufacturer's instructions.

S3 Figure. Plasma cytokines are not detectably increased by Western diet-feeding in *Apoe*$^{-/-}$ rats. Plasma cytokine levels (IL-1α, IL-4, IFNγ, CCL2 and TNFα) were measured three times during the study; when the animals were 8, 14 and 20 weeks of age. The plasma cytokines IL-1α, IL-4, IFNγ, CCL2 and TNFα were measured using the Rat Cytokine 5plex Kit FlowCytomix (#BMS826FF, eBioscience, Bender MedSystems GmbH, Vienna, Austria). The assay was performed according to manufacturer's instructions. Analysis was run as previously described [22] on a BD FacsCanto Flow Cytometer (BD Biosciences, Albertslund, Denmark) and processing of data was performed using the FlowCytomixTM Pro 2.3 Software (Bender MedSystems). Due to low sensitivity of the commercial kit used, the majority of data fell below detectable limit. With the inclusion criteria of minimum half of the samples being above detection limit within every group at a given time, it was only possible to analyse IL-1α (**A**) and CCL2 (**B**). Due to lack of Gaussian distribution data were analysed using the non-parametric Kruskal-Wallis test. No significant group differences were demonstrated (n=16 in the LF group, n=14 in the LF+G group, n=13 in the WD group and n=8 in the WD+G group).

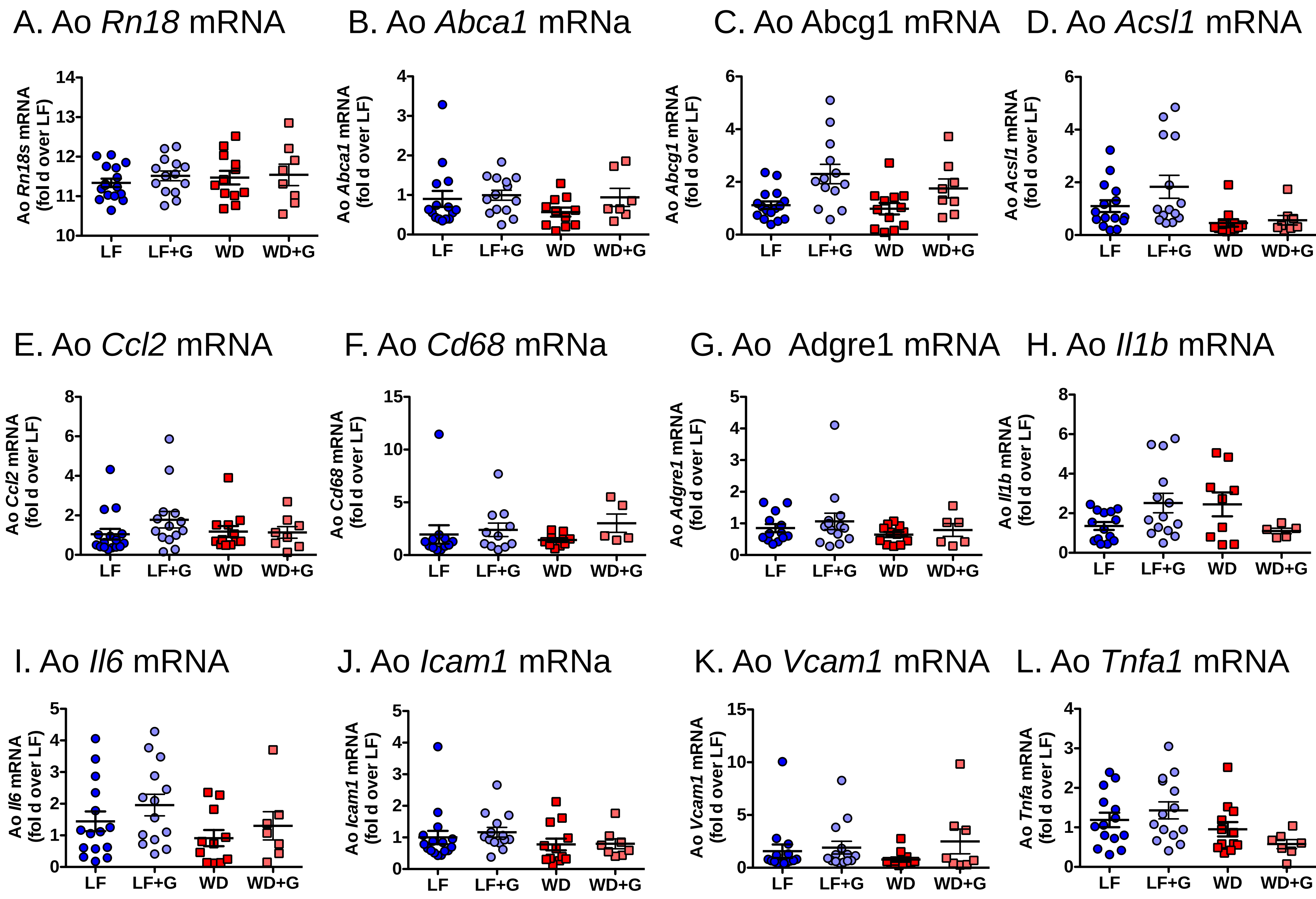
A. Ao Rn18 mRNA
Ao Rn18s mRNA (fol d over LF)
14
13
12
11
10
LF
LF+G
WD
WD+G
B. Ao Abca1 mRNa
Ao Abca1 mRNA (fol d over LF)
4
3
2
1
0
LF
LF+G
WD
WD+G
C. Ao Abcg1 mRNA
Ao Abcg1 mRNA (fol d over LF)
6
4
2
0
LF
LF+G
WD
WD+G
D. Ao Acsl1 mRNA
Ao Acsl1 mRNA (fol d over LF)
6
4
2
0
LF
LF+G
WD
WD+G
E. Ao Ccl2 mRNA
Ao Ccl2 mRNA (fol d over LF)
8
6
4
2
0
LF
LF+G
WD
WD+G
F. Ao Cd68 mRNa
Ao Cd68 mRNA (fol d over LF)
15
10
5
0
LF
LF+G
WD
WD+G
G. Ao Adgre1 mRNA
Ao Adgre1 mRNA (fol d over LF)
5
4
3
2
1
0
LF
LF+G
WD
WD+G
H. Ao Il1b mRNA
Ao Il1b mRNA (fol d over LF)
8
6
4
2
0
LF
LF+G
WD
WD+G
I. Ao Il6 mRNA
Ao Il6 mRNA (fol d over LF)
5
4
3
2
1
0
LF
LF+G
WD
WD+G
J. Ao Icam1 mRNa
Ao Icam1 mRNA (fol d over LF)
5
4
3
2
1
0
LF
LF+G
WD
WD+G
K. Ao Vcam1 mRNA
Ao Vcam1 mRNA (fol d over LF)
15
10
5
0
LF
LF+G
WD
WD+G
L. Ao Tnfa1 mRNA
Ao Tnfa mRNA (fol d over LF)
4
3
2
1
0
LF
LF+G
WD
WD+G

S1 Figure

Hemoglobin A1C

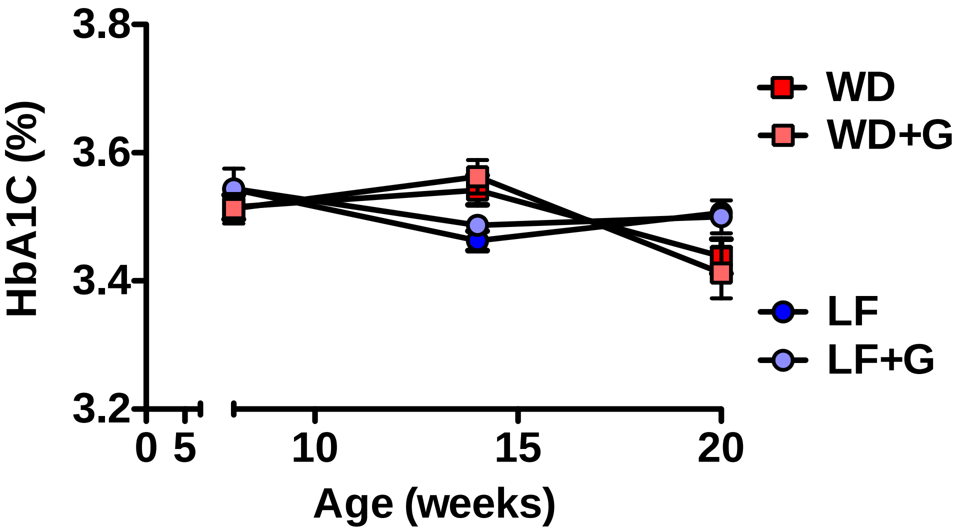

S2 Figure

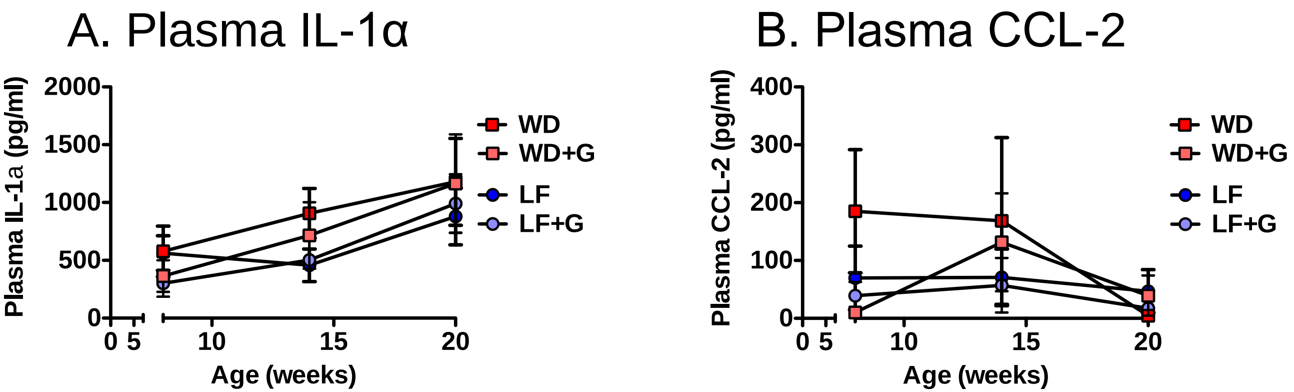
A. Plasma IL-1α
Plasma IL-1α (pg/ml)
2000
1500
1000
500
0
0 5 10 15 20
Age (weeks)
WD
WD+G
LF
LF+G
B. Plasma CCL-2
Plasma CCL-2 (pg/ml)
400
300
200
100
0
0 5 10 15 20
Age (weeks)
WD
WD+G
LF
LF+G

S3 Figure
